# Optimization of Sample Points for Monitoring Arable Land Quality by Simulated Annealing while Considering Spatial Variations

**DOI:** 10.3390/ijerph13100980

**Published:** 2016-09-30

**Authors:** Junxiao Wang, Xiaorui Wang, Shenglu Zhou, Shaohua Wu, Yan Zhu, Chunfeng Lu

**Affiliations:** 1 School of Geographic and Oceanographic Sciences, Nanjing University, Nanjing 210046, China; njuwjx@outlook.com (J.W.); njuwxr@163.com (X.W.); wsh@nju.edu.cn (S.W.); 2 Nanjing Nanyuan Land Development and Utilization Consulting Co. Ltd., Nanjing 210008, China; nyzhuyan@163.com (Y.Z.); lucff@163.com (C.L.)

**Keywords:** land evaluation, simulated annealing, soil sample points

## Abstract

With China’s rapid economic development, the reduction in arable land has emerged as one of the most prominent problems in the nation. The long-term dynamic monitoring of arable land quality is important for protecting arable land resources. An efficient practice is to select optimal sample points while obtaining accurate predictions. To this end, the selection of effective points from a dense set of soil sample points is an urgent problem. In this study, data were collected from Donghai County, Jiangsu Province, China. The number and layout of soil sample points are optimized by considering the spatial variations in soil properties and by using an improved simulated annealing (SA) algorithm. The conclusions are as follows: (1) Optimization results in the retention of more sample points in the moderate- and high-variation partitions of the study area; (2) The number of optimal sample points obtained with the improved SA algorithm is markedly reduced, while the accuracy of the predicted soil properties is improved by approximately 5% compared with the raw data; (3) With regard to the monitoring of arable land quality, a dense distribution of sample points is needed to monitor the granularity.

## 1. Introduction

Arable land is the basis of food production, the most valuable input in agricultural production, and an important factor in sustainable agricultural development and national food security [1,2]. In recent years, China has experienced rapid economic and social development. Among various issues, the reduction and degradation of arable land due to industrialization and urbanization has gradually emerged as one of the most prominent challenges in China [3,4,5]. In this context, the long-term dynamic monitoring of arable land quality becomes important for protecting arable land resources. With regards to the monitoring and evaluation of the arable land quality, several relatively complete networks of sample points have been established, and numerous soil data have been accumulated by the departments of land and resources and the departments of agriculture [6]. One efficient practice is to select representative sample points with the goal of achieving a certain level of accuracy [7,8,9,10]. However, little consideration has been given to optimizing their number and layout in previous monitoring studies on arable land quality [11,12]. Although spatial data sampling has been used in some regions [13,14,15], existing research based on spatial sampling theory has primarily involved spatial sampling that is optimized to focus on a particular indicator or property. Because the arable land quality involves various soil properties and land uses, it is difficult to accurately characterize the arable land quality using a single indicator [16,17]. Additionally, different indicators vary in their spatial effectiveness. Thus, when considering the optimization of sample points, various strategies are needed, depending on the indicators [18]. In addition, the distribution of soil properties displays spatial variations. However, existing sampling studies have paid little attention to spatial variations during scenarios with multiple indicators [18]. Therefore, it is necessary to further investigate how to improve the efficiency and accuracy of arable land quality monitoring and evaluation by optimizing the number and layout of sample points when there are spatial variations in multiple indicators [19].

Simulated annealing (SA) is a generic probabilistic algorithm for finding the optimal solution in a large search space [20]. The SA concept is derived from the principles of the annealing of solid materials from a certain initial temperature, with the temperature falling, for a combined probability jumping property. Based on that premise, the resulting algorithm is used to find the global objective function of the optimal solution in the solution space, which can be the local optima that jump out probabilistically and eventually become the global optima [21]. However, little consideration has been given to spatial variation in the use of the conventional SA algorithm. If the soil properties exhibit high spatial variation in a particular region, more sampling points should be located in this region. By contrast, if the soil properties exhibit low spatial variation, fewer sample points may be sufficient. For more sensitivity to changes in the space and area properties to focus on fine sampling, layout samples can be more appropriate; and for those places in which the spatial variation is insensitive and low, the sampling may be relatively lower. This decrease can save manpower and resources, and it can be used to acquire better soil attribute space distribution information [22]. In this study, spatial variation in soil properties was introduced as a new parameter to improve the use of SA. 

In the present study, Donghai, which is a typical agricultural county in the Huang-Huai-Hai Plain, was selected as the study area. The number and layout of soil sample points were optimized using a SA algorithm. The objectives of this study were: (1) to optimize the sampling strategy using SA while maintaining a certain level of accuracy and to monitor and evaluate the arable land quality accurately using fewer sample points; (2) to optimize the sample points with regards to multiple indicators of arable land quality and to analyze the characteristics and differences in sample layouts with regards to various indicators; (3) to improve the SA algorithm by considering the spatial variations in soil properties and then optimizing the number and layout of soil sample points; and (4) to investigate a method that reduces the number of sample points for characterizing the spatial distribution of soil properties while ensuring its accuracy, and to determine the optimal layout of sample points to achieve the highest accuracy. The minimum number and optimal locations for soil sample points are obtained by comparing the number and layout of sample points before and after optimization. 

## 2. Materials and Methods

### 2.1. Materials

#### 2.1.1. Study Area

**Figure 1:**
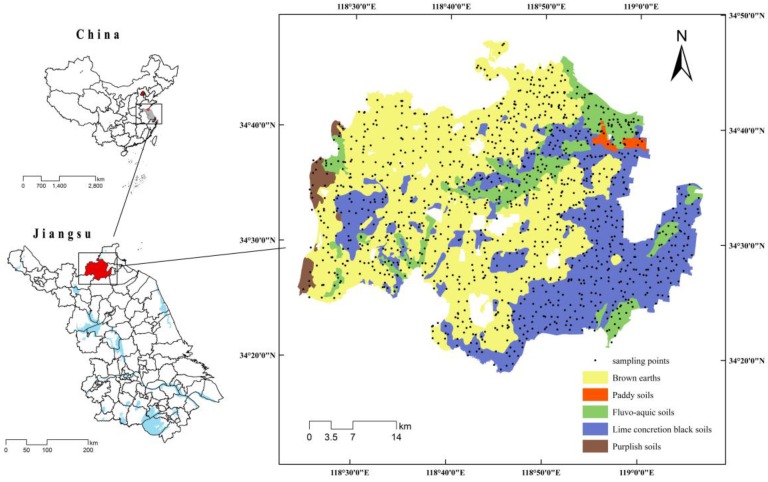
Location of the study area and sample points.

The county of Donghai (34°11′–34°44′ N, 118°23′–119°10′ E) is located in northeastern Jiangsu Province, China. As shown in Figure 1, Donghai is located in the Pingyuangang area on the southeastern edge of the Huang-Huai-Hai Plain. The plain generally slopes to the east. The eastern portion of the plain is flat and contains abundant lakes and reservoirs; the western hilly region is undulating and contains few water bodies; the central gently sloping region marks a transition from the plain to the hills. Donghai County is characterized by complex topography and is rich in soil resources that vary in profile structure and morphology, basic properties, and soil types. The soil types are diverse and include 17 soil genera, 46 soil species, and seven varieties. The land area of Donghai measures 200,981.02 ha, of which 77.44% is agricultural land, 18.30% is urban land, and 4.26% is unused land. The agricultural land represents 122,482.29 ha of the arable land (i.e., 60.948% of the total land area). Donghai has a high proportion of agricultural land and abundant arable land that is suitable for growing rice, wheat, corn, and other crops. Owing to the long history of agricultural production and intensive farming, land-use constraints have been greatly improved, and land productivity is relatively high. Reserve land resources are limited, and there is a lack of unused land that can easily be reclaimed following years of development, reclamation, and consolidation.

#### 2.1.2. Soil Sample Points

A total of 1440 sample points were randomly selected throughout the study area (Figure 1) after the rice harvests in November 2007, November 2008, and November 2009. Among them, 140 sample points were randomly selected as a validation set; the remaining 1300 sample points were included in the optimization by SA. Soil samples were collected from the 0–20 cm depth interval of the topsoil using a stainless steel soil sampler. At each point, five soil samples were collected in a square with a diagonal of 10 m, and the samples were uniformly mixed. One kilogram of soil that was taken by quartering was placed in a plastic bag and transported to the laboratory. The soil samples were air-dried before the laboratory analyses for organic matter and particle-size distribution {i.e., the fractions of clay, silt (coarse, medium, and fine), and sand (coarse, medium, and fine, ISO/CD11277)}. The soil pH was measured in situ. The locations of the sample points were recorded in terms of their geographic coordinates using a Garmin-76 GPS receiver (Garmin Corporation, New Taipei City, Taiwan) to improve accuracy with regard to the global positioning system. We focused on three indicators, namely the amount of soil organic matter, which served as a comprehensive indicator of arable land quality; the pH, representing the soil chemical properties; and the granularity, representing the soil physical properties. Extensive studies have shown that soil organic matter, pH, and granularity can account for more than 90% of the variance in arable land quality in the East China Plain [23,24]. Therefore, these three soil properties were selected as the indicators of arable land quality in the present study.

### 2.2. Methods

#### 2.2.1. Spatial Variation Analysis

Spatial autocorrelation analysis is one of the important methods for analyzing the spatial distribution of variations in soil properties [15]. The optimal layout of sampling points for monitoring arable land quality does not necessarily consist of a uniform distribution because of the different spatial variations in soil properties; rather, it is determined strategically and hierarchically depending on the spatial variations in soil properties [25]. Priority should be given to sampling for the properties and regions that are sensitive to spatial variations, which may require a relatively dense layout of sample points. By contrast, sampling can be relatively sparse with regard to properties and regions that are insensitive to spatial variations, which can conserve human and material resources while providing good information regarding the spatial distribution of the soil properties. Moran’s I is used to determine whether the phenomena or parameters are spatially aggregated. In contrast, local spatial autocorrelation yields the spatial variation of a particular geographic phenomenon or parameter within a small, local area. It is used to predict the spatial location and extent of aggregation at a site, and local indicators are used to determine the level of spatial autocorrelation between regional units [26,27,28]. The Moran’s I can be obtained using the following formula:

(1) $$I_{i}=n\left(x_{i}-\bar{x}\right)\overset{m}{\underset{j=1}{\sum }}w_{\mathrm{ij}}\left(x_{j}-\bar{x}\right)/\overset{n}{\underset{i=1}{\sum }}\left(x_{i}-\bar{x}\right)$$

where the value *w_ij_* is the weight assigned to areas *i* and *j*, and *x* is the property value of each sample.

#### 2.2.2. Spatial Variation-Based Simulated Annealing (SA) Algorithm

The SA algorithm is commonly used to optimize a sampling layout [20,21]. The use of conventional SA has been described by Chimi-Chiadjeu and other researchers [29,30,31]. The SA algorithm process of this paper is as follows: 

(1) A set for the initial solution was randomly selected from a maximum solution set as the optimal solution, which was used to calculate the corresponding objective function values *f*_0_. In this case, the maximum solution is the 1300 soil samples and the *f*_0_ is the root mean square error (RMSE).

(2) $$\mathrm{RMSE}=\sqrt{\frac{1}{n}\overset{n}{\underset{i=1}{\sum }}{\left(S_{i}-C_{i}\right)}^{2}}$$

where *n* represents the sample number validation set, which is 40. *S_i_* and *C_i_* represent the measured sampling point properties and predicted properties, respectively.

(2) Make random changes to the optimal solution to generate a new solution. In this paper, one point was randomly selected from the complementary set and it replaced a point from the initial solution to generate a new solution. The corresponding objective function value *f*_1_ was calculated, and then used in ∆ = *f*_1_ − *f*_0_. 

(3) If ∆ ≤ 0, then we accepted the new solution and replaced the current optimal solution; if ∆ > 0, then we accepted the new solution with a Metropolis criterion with probability *P*, otherwise keeping the original solution. The formula for calculating probability *P* is as follows:

(3) $$P=\frac{1}{1+exp\left(f_{1}-f_{0}\right)}>\delta$$

(4) Upon repeating steps (2) and (3) K times, it was determined whether the termination condition was satisfied. If not, the procedure was returned to step (2), or otherwise the output of the optimal solution was terminated. Algorithm termination conditions are selected to achieve the lowest RMSE.

Generally, the improved SA algorithm is still made up of four steps. The change is in step (2). In common SA, we randomly select one point from the complementary set. In improved SA, we select the point for which spatial variation is the maximum from the complementary set and replace a point from the initial solution with it to generate a new solution. The other parts remain unchanged.

An overlay analysis was conducted on the raw sample points and the spatial variation partitions of the three soil properties, which yielded the spatial variation at each sample point in a particular region. The Moran’s I index of each sample was obtained in ArcGIS 10.2 (Esri, Redlands, CA, USA) using the Cluster and Outlier Analysis toolbox. The resulting spatial variations provided by the sample points were used to improve the SA algorithm, and the root mean square error (RMSE) was used for the objective function.

#### 2.2.3. Statistical Analysis

The significance of differences between the results of the conventional and improved SA treatments within same soil property were determined by analysis of variance (ANOVA). The conventional and improved SAs were performed 10 times, and then a two-sample *t*-test was applied to compare the mean number of the sample points.

## 3. Results

### 3.1. Spatial Variation Partitions

**Figure 2:**
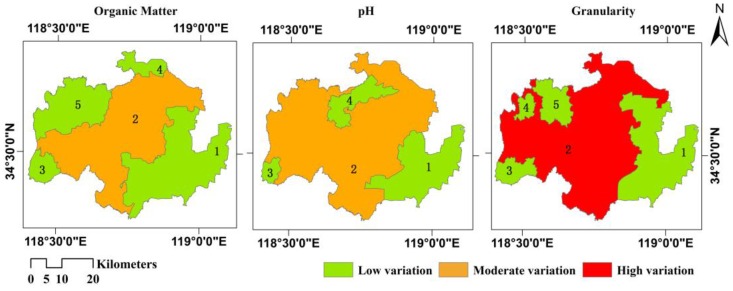
Spatial variation partitions of the three soil properties.

The spatial variation partitions of the three soil properties in the study area are depicted in Figure 2. The study area is divided into five partitions based on spatial variations in the amount of soil organic matter; this property is one of low variation based on the spatial autocorrelation analysis mentioned in the preceding section. Its low-variation partition, which represents 51.89% of the study area, is distributed over the eastern and western portions of the study area; the moderate-variation partition is distributed throughout the central region. The study area was also divided into four partitions based on variations in soil pH, which is also a low-variation property. The low-variation partition, which represents 27.16% of the study area, is scattered across the study area but is primarily found in the eastern portion; the remaining regions belong to the moderate-variation partition. The study area was then also divided into five partitions based on the variation in granularity, which is a moderate-variation property. Its high-variation partition, representing 64.45% of the study area, is distributed primarily around the central and western portions; the remainder belongs to the low-variation partition. The topography is complex and there are various soil types in the central and western regions of the study area, and the soil texture there displays marked spatial variations.

### 3.2. Conventional and Improved Simulated Annealing (SA)

The 1300 raw sample points of the soil organic matter, pH, and granularity were optimized using conventional and improved SA and Matlab software. The reduction in sample points using SA is compared with regard to the three soil properties in Table 1. Among the three soil properties, the granularity results in the largest number of sample points after the optimization using SA; the amount of organic matter ranks second. This result indicates that the soil granularity and organic matter require more sample points to reflect the distribution of the raw data, with a relatively narrow range in the number of effective sample points. The SA optimization results in no more than 100 sample points for the soil pH, indicating that this soil property requires fewer points to reflect the characteristics of the raw data, with a relatively broad range in the number of effective sample points. This difference is primarily due to the different spatial variations among the soil properties, which require different numbers of sample points to express them. With the improved SA, the number of optimal sample points is further reduced, and the ranges of effective numbers of sample points for all three soil properties are broadened. This finding is explained in that the improved SA is more targeted in selecting the sample points. It selects more sample points in high-variation regions and fewer sample points in low-variation regions. This selection approach can achieve good monitoring results with fewer sample points. 

**Figure 3:**
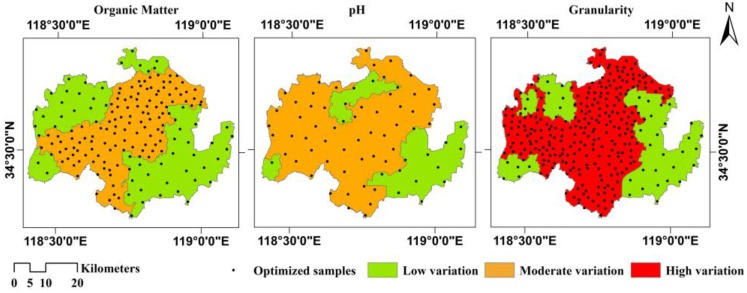
Spatial distribution of sample points for the three soil properties after optimization with the improved simulated annealing (SA).

The improved SA results in 178, 72, and 315 optimal sample points for monitoring the soil organic matter, pH, and granularity, respectively. The optimal spatial distribution of sample points for the three soil properties is illustrated in Figure 3, which shows a specific pattern. The sample points are densest in the central and western regions and relatively sparse in the eastern region. This improved sample distribution closely coincides with the spatial variation in each soil property. The highest spatial variation is located in the central region, where the distributions of sample points for all soil properties are most dense; the western region ranks second; and the eastern region shows the lowest spatial variation, where the improved sample distribution is relatively sparse. This pattern suggests that the distribution of sample points obtained with the improved SA can effectively reflect the spatial variation in regional soil properties.

The western region is characterized by low hills, generally low levels of soil organic matter and granularity, and neutral to slightly acidic soil pH values. The eastern region is characterized by plains, generally high levels of soil organic matter and granularity, and slightly alkaline soil pH values. The central region is characterized by gentle slopes at the transition from the plains to the hills, complex topography, diverse soil types, and dramatic spatial variations in soil properties. Therefore, it is necessary to locate more sample points in the central region to reflect the spatial variation in the soil properties fully. These results indicate that the distribution of sample points obtained with the improved SA is consistent with the topographic features, and the optimized sampling for these soil properties is appropriate for the monitoring and evaluation of local arable land quality.

In summary, there is a clear hierarchy in the spatial distribution of sample points for the three soil properties after optimization using the improved SA. The optimal sample points reflect the spatial variation in soil properties and comply with requirements for the monitoring and evaluation of arable land quality based on these soil properties. The improved SA based on the spatial variation in soil properties is superior to a spatial distribution of sample points that neglects this variation.

Table 2 presents descriptive statistics for the three soil properties based on the optimal and raw sample points. The means of the three soil properties show small differences between these two sets of points. These differences are no greater than 7% for the organic matter, pH, and topsoil thickness and 6.45% for the granularity, without significantly affecting the expression of the soil texture. For the coefficient of variation (CV), the three soil properties display very small differences between the two sets of sample points, suggesting that the optimal sample points obtained with the improved SA reflect the variation in the raw data. Therefore, based on the spatial distribution of sample points and the descriptive statistics of the data, the optimization of soil sample points with the improved SA can effectively reduce the number of sample points while retaining the variation in the raw data. This finding indicates that it is rational to improve the SA algorithm and to optimize the number of soil sample points for the monitoring and evaluation of the arable land quality.

## 4. Discussion

### 4.1. Comparison of Optimal and Raw Soil Sample Points

Table 3 lists the soil properties obtained from the raw sample points and from the two SA algorithms. For all three soil properties, the improved SA yields a smaller number of optimal sample points than the conventional SA. Greater numbers of sample points resulting from the conventional SA correspond to greater reductions in the number of sample points with the improved SA. For the soil pH, the number of sample points is reduced from 78 to 72 when using the improved SA (i.e., a reduction of 6 points). For the soil granularity, the number of sample points is reduced from 418 to 315 when using the improved SA (i.e., a reduction of 103 points). The improved SA captures the variation in the data as well as the raw set of sample points but uses fewer sample points. This improved algorithm therefore yields a more efficient set of sample points.

We obtained the mean local Moran’s I index as the spatial variation in soil properties in each region based on the raw sample points, optimal sample points from the conventional SA, and optimal sample points from the improved SA by performing an overlay analysis of the spatial variation partitions of soil properties and these three sets of sample points. The results indicate that the mean spatial variations in soil properties based on the optimal sample points from the conventional SA differ slightly from those of the raw sample points; the values for the soil organic matter and pH are smaller, and the optimal sample points from the conventional SA are selected according to their statistical characteristics. The layout of these sample points is not targeted to the objectives of the study but instead display a type of blindness. The mean spatial variation in soil properties is markedly higher with the optimal sample points from the improved SA compared with the other two groups. This result suggests that in addition to the statistical characteristics of the sample points, the improved SA gives full consideration to the spatial variation in regional soil properties in the selection of sample points. The optimization of sample points with the improved SA is therefore clearly selective and targeted.

Therefore, in the optimization results based on the soil properties, the sample points that were optimized with the improved SA better reflect the statistical characteristics of the raw data and the spatial variation in soil properties, regardless of the spatial distribution of sample points or the RMSE of the optimization results and the probability of selection.

Based on the spatial variation partitions, the optimization of sample points in various partitions regarding each soil property is summarized in Table 4. 

As shown in Table 4, the improved SA produces different results when optimizing the sample points in different spatial variation partitions. Specifically, more sample points are retained in the moderate- and high-variation partitions, whereas fewer sample points are retained in the low-variation partitions. The optimal sample points represent 17.94% of the raw sample points in the case of soil organic matter in the moderate- and high-variation partitions and 9.19% of the points in the low-variation partition. The effect is not evident in the sample points for soil pH, and their corresponding percentages are 5.81% and 4.9%, respectively. The most significant effect is observed among the sample points for soil granularity, for which the percentages are 33.05% and 9.47%, respectively (a 23.58% difference). Combined with the data characteristics of the raw sample points, these results indicate that higher CVs for soil properties lead to greater differences in the optimization of sample points between high-variation and low-variation partitions. Among the three soil properties, the CV is lowest for the pH and highest for the granularity. Therefore, the numbers of optimal sample points for the soil pH in the low- and moderate-variation partitions are nearly equal, whereas the optimal sample points for the soil granularity tend to be located in the moderate- and high-variation partitions. More sample points are needed to ensure high accuracy in regions with relatively high spatial variations in soil properties.

### 4.2. Prediction Accuracy of Arable Land Quality Based on Optimal Soil Sample Points

The prediction accuracy of optimal sample points for soil properties, which was obtained using conventional and improved SA, was quantitatively analyzed with the validation set of 140 sample points collected in this study. The sample points for the three soil properties that were optimized using the two SA algorithms were subjected to Kriging interpolation. The interpolated results were fit linearly to 40 verification points. The R^2^ of the fitting equation was used to indicate the accuracy of the predicted value with respect to the measured value, thereby providing a quantitative assessment of the prediction accuracy of the sample points that were optimized using the two SA algorithms. Furthermore, the raw sample points and the optimal sample points obtained using the two SA algorithms were compared to address the accuracy of the predicted soil properties, as shown in Table 5.

The accuracy of the predicted soil properties based on the optimal sample points from the conventional SA is lower than that of the raw sample points. However, the optimal sample points from the improved SA yield accurate predictions of the soil properties; this accuracy is significantly higher than the one that is based on the conventional SA and slightly higher than that based on the raw sample points. Moreover, the number of optimal sample points obtained with the improved SA is markedly less than that obtained with the conventional SA and far less than the number of raw sample points, or 1300. However, the sample points obtained with the improved SA result in significantly more accurate predictions than those obtained with the conventional SA; the former also result in higher accuracy than the raw sample points. Therefore, the improved SA achieves more accurate predicted soil properties based on fewer optimal sample points. This result demonstrates that it is reasonable to optimize the number and layout of soil sample points using SA and that the modified SA presented in this study is useful. 

### 4.3. Analysis of Optimal Sample Points

**Figure 4:**
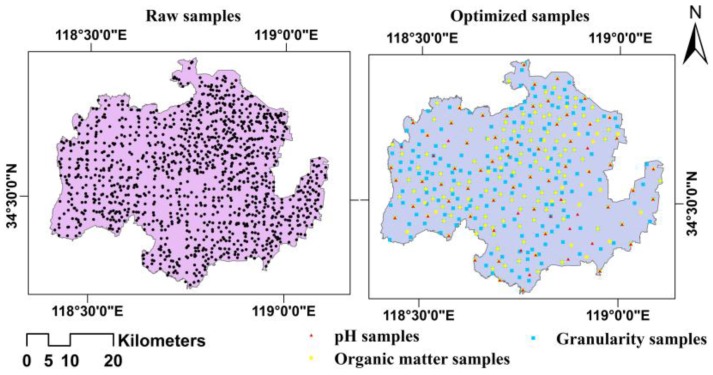
Spatial distribution of optimal sample points for the three soil properties in the study area.

The optimized sample points for the three soil properties were combined to obtain the spatial distribution of all the optimal sample points across the study area (*n* = 349, Figure 4). 

As shown in Figure 4, this optimization results in the greatest decrease in sample points in the eastern region, which is characterized by plains with soil properties of relatively low variation; thus, this region can be monitored with a small number of sample points. The western region, which is characterized by hills, ranks second. The smallest decrease in the number of sample points occurs in the central region, which is characterized by gentle slopes in the transition zone between the western hilly region and the eastern plain region. Owing to its high regional variation in soil properties, a dense layout of sample points is required to achieve a certain monitoring accuracy in the central region.

The numbers of optimal sample points after combining the assessment requirements are summarized in Table 6.

Table 6 shows a partial overlap between the optimal sample points for the three soil properties. In total, there are 349 optimal sample points, 45.27% of which are needed to monitor the soil granularity only. Thus, the soil granularity demands the greatest number of sample points in the study area. Additionally, 26.93% of the sample points are coincident points for monitoring both soil organic matter and granularity, and 13.18% of the sample points are coincident points for soil organic matter, granularity, and pH. Only 3.44% of the optimal sample points are used for soil pH only, and another 3.44% are for both soil pH and organic matter. The optimal sample points primarily target the monitoring of soil granularity, whereas the number of sample points required for monitoring the soil pH is markedly low. For the long-term dynamic monitoring of arable land quality, it is most important to monitor the soil granularity, followed by the amount of soil organic matter; the soil pH is the least important parameter to monitor in this area. 

## 5. Conclusions

We partitioned the study area based on the spatial variation in soil properties, and we improved the SA algorithm by introducing the spatial variation in soil properties into the SA procedure as a new parameter. We used the improved SA algorithm to optimize the number and distribution of sample points for three soil properties and then evaluated the prediction accuracy of the optimal sample points. The major conclusions are as follows:

(1) Despite a large reduction in the number of sample points, all three predicted soil properties retain the statistical characteristics of the raw data, and the optimal sample points are uniformly distributed in space. Compared with the conventional SA algorithm, the improved SA algorithm further reduces and optimizes the number of sample points, while all three properties retain the statistical characteristics of the raw data. During the optimization procedure, more sample points are retained in the moderate- and high-variation partitions, whereas fewer sample points are retained in the low-variation partitions. Higher CVs for soil properties lead to greater differences in the optimization of sample points between the high-variation and low-variation partitions. To ensure high monitoring accuracy, more sample points are needed in regions with relatively high spatial variations in soil properties.

(2) The improved SA achieves higher prediction accuracy for soil properties through the selection of fewer (optimal) sample points. The number of optimal sample points obtained from the improved SA is markedly reduced, while the accuracy of the predictions is improved by approximately 5% compared with the raw data. It is therefore reasonable to optimize the number and layout of soil sampling points using SA, and the modified SA developed in this study is useful. 

(3) A total of 349 sample points are obtained by combining the optimization of sample points for the various soil properties. To monitor the arable land quality, a dense set of sample points is required for monitoring the soil granularity, whereas monitoring the pH requires the lowest number of sample points in the study area. For the long-term dynamic monitoring of arable land quality, it is most important to monitor the soil granularity, followed by the soil organic matter; the soil pH is the least important parameter to monitor in this area.

## Figures and Tables

**Table 1:** Comparison of sample point optimization concerning the three soil properties using conventional and improved simulated annealing (SA).

**Conventional Simulated Annealing**						
**Indicator**	**Number of Optimal Sample Points**	**Percentage of Optimal Sample Points**	**Range of Effective Sample Points**	**Proportion of Effective Sample Points**	**RMSE**	**ANOVA**
Organic matter	226	17.38%	57–1245	4.38%–95.77%	0.49	**
pH	78	6.00%	35–1266	2.69%–97.38%	0.44	*
Granularity	418	32.15%	103–1274	7.92%–98.00%	15.54	**
**Improved Simulated Annealing**						
**Indicator**	**Number of Optimal Sample Points**	**Percentage of Optimal Sample Points**	**Range of Effective Sample Points**	**Proportion of Effective Sample Points**	**RMSE**	**ANOVA**
Organic matter	178	13.69%	52–1251	4.00%–96.23%	0.50	**
pH	72	5.54%	31–1273	2.38%–97.92%	0.44	*
Granularity	315	24.23%	88–1280	6.77%–98.46%	15.57	**

RMSE: Root mean square error; ANOVA: Analysis of variance; **: Extreme significant; *: Significant.

**Table 2:** Descriptive statistics of soil properties based on raw data and on sample points that were optimized with the improved simulated annealing (SA) algorithm.

Parameter	Organic Matter (g/kg)		pH		Granularity (%)	
	Raw Sample Points (*n* = 1300)	Optimal Sample Points (*n* = 178)	Raw Sample Points (*n* = 1300)	Optimal Sample Points (*n* = 72)	Raw Sample Points (*n* = 1300)	Optimal Sample Points (*n* = 315)
Minimum	1.2	6.6	5.0	5.0	3.5	3.5
Maximum	41.0	37.0	9.9	8.9	89.7	88.6
Mean	18.4	17.6	6.7	6.7	34.1	30.9
Skewness	0.51	0.8	0.35	0.96	0.61	1.26
Kurtosis	–0.3	0.38	0.77	1.16	−0.96	0.61
Coefficient of variation	37.33%	37.28%	8.64%	6.00%	69.32%	72.28%
ANOVA	**		*		**	

ANOVA: Analysis of variance; **: Extreme significant; *: Significant.

**Table 3:** Comparison of optimization results for different soil properties obtained using two simulated annealing (SA) algorithms.

Soil Property	Spatial Variation		
	Raw Sample Points	Optimal Sample Points from Conventional SA	Optimal Sample Points from Improved SA
Organic matter	0.3458	0.3412	0.4288
pH	0.4700	0.4553	0.4859
Granularity	0.4898	0.5001	0.6423

**Table 4:** Comparison of the optimization results for soil properties in different spatial variation partitions by improved simulated annealing (SA).

Soil Property	Low-Variation Partitions			Moderate- and High-Variation Partitions		
	Raw Sample Points	Optimal Sample Points	Percentage of Optimal Sample Points	Raw Sample Points	Optimal Sample Points	Percentage of Optimal Sample Points
Organic matter	631	58	9.19%	669	120	17.94%
pH	388	19	4.90%	912	53	5.81%
Granularity	486	46	9.47%	814	269	33.05%

**Table 5:** Accuracy of predicted soil properties based on sample points that were optimized using the two simulated annealing (SA) algorithms.

Soil Property	Raw Sample Points		Optimal Sample Points from Conventional SA		Optimal Sample Points from Improved SA	
	Number	Prediction Accuracy (R^2^)	Number	Prediction Accuracy (R^2^)	Number	Prediction Accuracy (R^2^)
Organic matter	1300	0.8823	226	0.8331	178	0.8926
pH	1300	0.7363	78	0.7108	72	0.7488
Granularity	1300	0.8527	418	0.8116	315	0.8693

**Table 6:** Numbers of optimal sample points.

Source	Number	Percentage
Optimal sample points for organic matter only	25	7.16%
Optimal sample points for pH only	12	3.44%
Optimal sample points for granularity only	158	45.27%
Optimal sample points for organic matter and pH	12	3.44%
Optimal sample points for organic matter and granularity	94	26.93%
Optimal sample points for pH and granularity	2	0.57%
Optimal sample points for organic matter, pH, and granularity	46	13.18%
